# Experiences and Unmet Needs of Adolescent and Young Adult Survivors of a Brain Tumor (Aged 15–39 Years): A Systematic Review and Meta-ethnography

**DOI:** 10.1097/NCC.0000000000001311

**Published:** 2023-12-08

**Authors:** Kate Law, Iram Salam, Martin G. McCabe, Sabine N. van der Veer, Faith Gibson, Janelle Yorke

**Affiliations:** Author Affiliations: Division of Nursing, Midwifery and Social Work, The University of Manchester (Ms Law and Dr Yorke); The Christie Hospital NHS Foundation Trust (Mss Law and Salam, and Drs McCabe and Yorke), Manchester; and Division of Cancer Sciences (Dr McCabe) and Centre for Health Informatics, Division of Informatics, Imaging and Data Sciences, Manchester Academic Health Science Centre (Dr van der Veer), The University of Manchester; Centre for Outcomes and Experience Research in Children’s Health, Illness and Disability, Great Ormond Street Hospital for Children NHS Foundation Trust, London; and School of Health Sciences, University of Surrey (Dr Gibson), Guildford, United Kingdom.

**Keywords:** Adolescents and young adults, Brain tumor, Meta-ethnography, Quality of life, Survivorship

## Abstract

**Background:**

Brain tumors account for 15% of all adolescent and young adult cancers, and survivors are at risk of ongoing late effects that can severely impact their ability to reach independence. Despite follow-up initiatives advocating a personalized approach, survivors continue to experience ongoing sequelae. A better understanding of the survivorship experience is required to ensure services are able to deliver personalized support.

**Objective:**

The aim of this systematic search and meta-ethnography was to identify and synthesize qualitative studies to better understand the experiences, perspectives, and needs of adolescent and young adult brain tumor survivors.

**Methods:**

Five databases were searched using predefined criteria, studies were independently screened by two researchers, and those meeting inclusion criteria were synthesized.

**Results:**

Twenty-seven studies were synthesized, generating 2 main themes, each with subthemes: (1) individual factors impacting resilience, namely, positive coping styles, managing emotions, and family functioning, and (2) cancer-related factors that challenge the individual, namely, living with societal expectations and barriers to coping.

**Conclusion:**

The conceptual framework illustrates the challenges and resilience of survivors along the continuum from adolescence to adulthood, reflecting the needs of this age group in 1 model, despite it being a time of rapid growth. The lack of awareness of potential physical and emotional late effects challenges individual resilience, which is further challenged when significant milestones cannot be reached.

**Implications for Practice:**

There is a role for follow-up services to identify and address unmet needs, provide better information to equip survivors to manage late effects, and support families, particularly those who underwent more intensive treatment.

Brain and central nervous system tumors are the most common solid tumor across 153 cancer registries worldwide for those aged 0 to 19 years.^1^ They account for 15% of adolescent and young adult (AYA) cancers aged 15 to 25 years.^2^ Adolescents and young adults aged 15 to 39 years have the highest rates of survival after a brain tumor, which when combined with the incidence rate in this age group, indicates this population has the largest proportion of survivors predisposed to late effects. More than half of survivors of a brain tumor have ongoing symptoms that can have a detrimental impact on an individual’s ability to attain common milestones reached during adolescence.^3^ Failure to achieve these milestones highlights the susceptible nature of adolescence and the vulnerability of those severely affected by their diagnosis and treatment. Lower rates of marriage and employment, social isolation, and limits on reaching independence are a consequence of late effects from disease and treatment.^3–6^

The aim of support for cancer survivors is to maximize quality of life by aiding a return to normality and independence as soon as possible while minimizing the negative effects associated with cancer and its treatment.^7^ Recommendations for follow-up services advocate and incentivize implementation of personalized packages of follow-up care composed of well-being events aimed to support self-management of symptoms, rapid access to a cancer center when necessary, completion of holistic needs assessments, and access to health and well-being information.^8^ In addition, survivors of a brain tumor should be made aware that lifestyle choices may impact on the severity and management of late effects. Therefore, follow-up services need to acknowledge typical behavior traits displayed in this age group: immature decision making and not placing emphasis on the long-term consequences of decisions.^9^ A better understanding of all aspects of the survivorship experience of AYAs is required to ensure follow-up services are able to meet the needs of this population.

Systematic reviews examining quality of life and experiences of brain tumor survivors mainly focus on adult^10,11^ or pediatric^12,13^ populations. This meta-ethnography aims to add to existing knowledge by aggregating qualitative data and interpretively synthesizing this to generate new concepts regarding all aspects of the survivorship experience. To identify areas of unmet needs for young people aged 15 to 39 years, the focus of this review is on quality of life, family functioning, experiences of physical and cognitive late effects, and social functioning, including the perspectives of parents, partners, and caregivers.

## Objectives

The primary objective was to identify and synthesize qualitative studies to better understand the experiences, perspectives, and needs of AYA brain tumor survivors (aged 15–39 years).

The secondary objectives were the following:

1) Explore the effect of time since diagnosis on the needs of young people as they progress through adolescence.
2) Examine how the type of brain tumor and/or treatment may impact the experience and needs of survivors.
3) Examine parental perceptions of the young persons’ needs.

## Methods

A systematic search strategy and meta-ethnography guided by the 7 phases outlined by Noblit and Hare^14^ was conducted, and the report follows France et al’s^15^ guidance to maximize transparency. Noblit and Hare^14^ suggested the following 7 steps: getting started, deciding what is relevant, reading the studies, determining how studies are related, translating studies, synthesizing translation, and expressing the synthesis. The research team consisted of clinicians and researchers with expertise in AYA oncology and meta-ethnography. Conducting a systematic review did not require ethical board approval.

### Search Strategy and Screening Process

A preplanned electronic search of published articles written in the English language between 2000 and 2021 was conducted using the population, exposure, outcome framework^16^ to identify search concepts related to survivors (population), brain tumors (exposure), and experience (outcomes) (see the Appendix, http://links.lww.com/CN/A177, for an example of search history in MEDLINE). The timeline of the search strategy reflects the establishment of AYA cancer services in the United Kingdom and internationally, as well as the implementation of national strategies to ensure provision of age-appropriate services for all young people.^17^ Databases from nursing, medical, and social science disciplines were searched to maximize the capture of the experience of survivors from all perspectives. We also hand-searched reference lists, Google Scholar, and specific prominent journals where information on survivorship is largely published (such as the *Journal of Adolescent and Young Adult Oncology* and *Journal of Pediatric Hematology/Oncology Nursing and Psycho-Oncology*).

### Inclusion and Exclusion Criteria

Two independent researchers (K.L. and I.S.) completed 2-stage screening of titles and abstracts of the retrieved studies according to the inclusion/exclusion criteria (Table 1).

**Table 1:** Inclusion/Exclusion Criteria

	Inclusion Criteria
1	Studies of qualitative nature (all methodologies) with adolescents and young adults aged 15–39 years published in peer-reviewed journals
2	Participants diagnosed as a child, adolescent, or young adult with a brain tumor of any type (including malignant and nonmalignant)
3	Completed active treatment
4	Personal narratives and individual commentaries published in peer-reviewed journals
5	Mixed-method studies where the qualitative data could be extracted
6	Studies including parent/caregiver perceptions of experience caring for a brain tumor survivor
7	Studies in adult (ie, including participants aged >39 y) or pediatric populations where quotes from participants within the age range of 15–39 years could be identified
8	Studies of mixed cancer populations where quotes from participants with a brain tumor could be identified
	**Exclusion Criteria**
1	Studies not written in the English language

The first stage of screening excluded those not meeting the inclusion criteria. Full-text screening was carried out in stage 2 and recording the reason for exclusion. Disagreements were resolved through discussion with a third member of the team (F.G.).

Where studies included populations with mixed cancer types, studies were included if quotes from survivors of a brain tumor could be identified and extracted. Similarly, where the study population range was outside 15 to 39 years, studies were included if quotes of participants within our defined age range were identifiable. This allowed an inclusive approach to ensuring all studies reporting qualitative data regarding the experiences of AYA survivors of a brain tumor aged 15 to 39 years could be included.

### Data Extraction

Study characteristics were documented in a summary data table to enable an understanding and comparison of the data. Contextual information such as the diagnosis and type of treatment were recorded with the participants’ quotes where available to allow exploration of the secondary objectives. In congruence with meta-ethnography,^18^ studies were not excluded because of quality or ability to produce rich data (which reveals the complexities of what is being studied).^14^ Quality assessment was carried out by 2 independent researchers (K.L. and I.S.) and judged the ability of a study to produce higher level analysis that goes beyond a narrative. Criteria by Walsh and Downe^19^ were used to make an objective decision regarding quality (yes, no, unclear) and are reported in the summary data table with examples to support decisions made.

### Data Synthesis

Data synthesis involves systematically comparing studies to identify similarities and differences between the included studies, known as reciprocal and refutational translation, respectively. Quotes, known as first-order data, from each study can be used as evidence to support reciprocal translation between studies, where similarities exist.^14^ Where quotes oppose the reciprocal translation, or report contradictory information, this is an example of refutational translation. Data synthesis consists of the construction of themes (third-order data) that incorporate reciprocal and refutational translation. For this meta-ethnography, we analyzed first- and second-order data and the original authors’ concepts together rather than in isolation to generate the new line of argument and conceptual framework.^17^ Codes were developed to label first-order data, which were taken from the results section of each article, during an iterative process of reading, rereading, and interpretation to allow the researchers to determine how studies were related. Codes were then tabulated to allow data to be compared and contrasted, allowing the translation phase of synthesis (Table 2). Similar codes were grouped into concepts. Two researchers (K.L. and I.S.) completed this stage to minimize bias when allocating codes to data.

**Table 2:** Examples of Codes With Contextual Information Where Available

Concept	Code	Supporting Quotes	Contextual Information About the Participant	
			Diagnosis	Treatment
Positive coping styles	Optimism	“Throughout my life, basically it all, and in grade school, kindergarten, high school, middle school, basically just some things were challenging for me. But I tried to make the best out of it.”^20^	Medulloblastoma	Chemotherapy, surgery, radiotherapy
Positive coping styles	Positive coping	“We don’t focus on the condition, it used to be the first thing, that is not how life works, life is hard for all kids at one point.”^21^	Medulloblastoma	Chemotherapy, surgery, radiotherapy
Managing emotions	Loss of old life	“She gets frustrated because of the things she could do that she can’t do now. She was very sporty, she was always running and riding her bike and they’re the things she can’t do now” (parent).^22^		
Managing emotions	Poor self-esteem	“Sometimes you feel like your value to your family or society is not as good as the person next to you. I’m not making any contribution to society by going there and working. You feel less of a person because you can’t go there.”^23^		

Multiple discussions between 3 members of the research team (F.G., K.L., and I.S.) allowed themes to be developed through synthesizing and identifying reciprocal or refutational codes. The final stage of analysis was to draw conceptual maps illustrating relationships between themes and demonstrate synthesizing translations. Discussion and translation of themes enabled us to use our third-order data to develop a new line of argument, forming a conceptual framework regarding the ongoing daily experience of surviving a brain tumor. The framework was developed through 5 iterations of the concept map.

## Results

**Figure 1:**
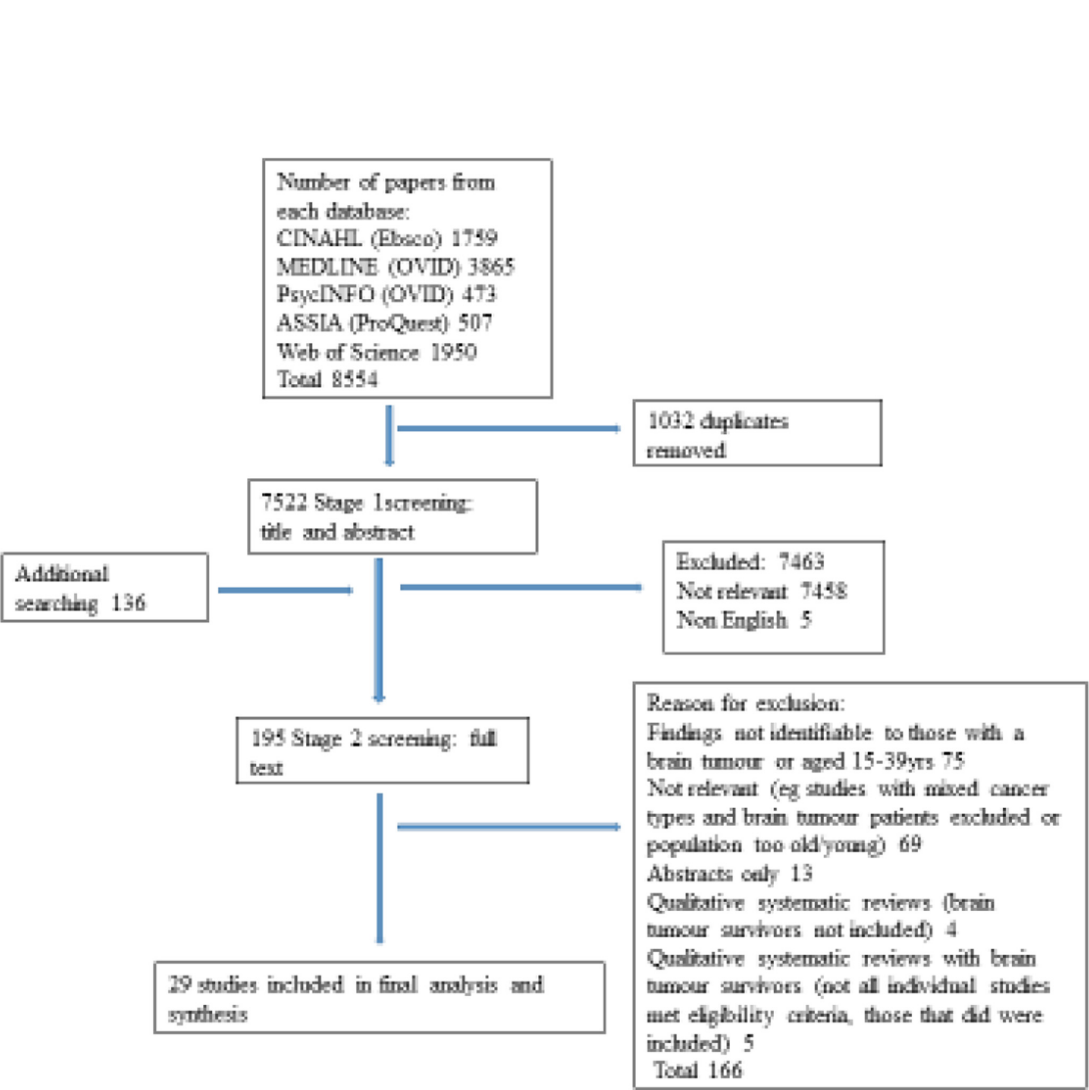
Search and screening process (last run, August 3, 2023).

Figure 1 highlights the search and screening process. Study characteristics and themes were documented and included in the synthesis.

## Study Characteristics

Twenty-nine studies from 7 different countries met the inclusion criteria. Table 3, http://links.lww.com/CN/A232, summarizes the study characteristics, main findings, and critical appraisal.

Two studies included mixed cancer types where quotes from survivors of a brain tumor could be identified.^24,25^ The review included studies representing different life experiences of survivors such as family functioning, cognitive late effects, health-related quality of life (HRQOL), and education or employment. One article was a personal narrative of one parent’s experience of caring for a childhood survivor of a brain tumor^26^ and 2 articles were reflections from a nurse’s experience of joining a peer support group attended by survivors of brain tumors.^27,28^

### Themes

Reciprocal and refutational translation of studies revealed concepts relating to the individuals’ experience of life after treatment of a brain tumor, which were represented across the continuum from adolescence to adulthood. We were able to address our objectives, and each theme incorporates the effect of time since diagnosis, the impact of different types of tumor and treatment on these experiences, and parental perceptions where possible. Two themes and 5 subthemes are reported and represent aspects of survivors’ lives. The first theme related to the individual, and subthemes were seen as integral to the daily life of survivors of a brain tumor. The second theme related to the impact of cancer, treatment, and its late effects on the person.

### Individual Factors Impacting the Resilience of Survivors

#### Positive Coping Styles

The individual nature of coping styles was apparent from reciprocal translation of all included studies regardless of age, diagnosis, or severity of late effects, and many survivors and caregivers were able to find meaning and positivity from their experience, portraying a strong determination and drive to achieve their goals^23,28,29,32^:

The doctors tell me I would never graduate high school…and I was like, yeah, I'm showing you…I was able to do it.^29^

Other positive attitudes continued to emerge throughout studies regardless of the age or diagnosis of the survivor. Where positive ambition and ability to adjust exist, young people were able to maintain some control over their situation.^20,21,27,29,31,36,40,43^

Positive attitudes were evident by young people’s reflections on their situation:

After everything I've been through, I feel like superman.^20^

The concept of feeling like a survivor had both positive and negative implications, demonstrating refutational analysis within this theme. Some felt they did not deserve the title of survivor, whereas others felt the term *survivor* labeled a traumatic event and kept the diagnosis present. In addition to the internal drivers of positive coping, having a faith contributed to making sense and maintaining control of the situation:

God picked me for a reason. He picked me to teach him how to deal with this.^34^

Individual coping styles were apparent throughout the studies, representing individual resilience, and were reported by participants regardless of the severity of disease/treatment or age. However, the ability to remain positive was conflicted with the presence of different emotions expressed by individuals.

#### Managing Emotions

Despite the ability to maintain positivity, reciprocal translation of studies also found survivors of all ages portraying a range of negative emotions such as persistent anxiety, depression, and suicidal thoughts, adding a burden on survivors and their families.^22,23,29,31,40^

I didn’t want to tell my parents I was injured by my classmates, so I banged the wall to hurt my hands in my bedroom and considered suicide.^31^

Feelings of anger and frustration spanned the types of disease and treatment, although they were felt to a greater extent in those whose late effects were more limiting. Feelings of inadequacy and poor self-esteem due to perceiving themselves as a burden on the family were also reported:

Sometimes you feel like your value to your family or society is not as good as the person next to you. I’m not making any contribution to society by going there and working. You feel less of a person.^23^

A sense of loss was common to survivors: loss of the life planned/old life, loss of friendships, and loss of physical function that impacted on a survivor’s day to day.^22,23,26,38,39,46^ Many survivors and caregivers were scared of the unpredictable nature of cancer and described fear of recurrence or fear for their future.^22,27,35,38,39,41,43,46^

I don't ever relax.. If you get too comfortable that’s when things will happen.^41^

It is clear from the literature that survivors continue to experience a lasting range of emotions that can help or hinder their day-to-day life and persistent late effects influence the number of negative emotions being expressed.

#### Family Functioning

The significance of support from family members from adolescence into adulthood was acknowledged as being crucial throughout this continuum and studied by many.^20–22,41,44–46^ Refutational analysis identified the change in the definition of family depending on the individual’s place along the continuum from adolescence into adulthood. Significant others were parents and siblings at the younger end of this spectrum, whereas partners/spouse took this place for older individuals. Families who are able to remain “family focused” have a greater ability to adapt, incorporate management of any conditions into family life, and report higher HRQOL compared with families who were “condition focused.”^20,44^ The ability to remain “family focused” was directly related to treatment intensity, and where families were condition focused, the survivor was more likely to have experienced intense treatment and severe late effects. Where the impacts are ongoing, there is a constant feeling of anticipation^21,44–47^:

I’d like to say that her condition is not the most important thing in our family, but there’s not a day that goes by that I don’t think about it. Either I am thinking about the late effects or the chance of it coming back. It’s like waiting for the other shoe to fall.^21^

Family functioning was also described by Hammond^26^ and Lucas et al^40^ who demonstrated higher levels of anxiety and uncertainty were associated with increased caregiver demands. Where survivors had a greater dependence on their family, they also relied on them for their main source of social support^47^:

My mother in particular is my backbone.^20^

Survivors felt a burden on their family across the continuum of adolescence into adulthood. Younger adolescents recognized how much their parents did for them and wanted to return their help by doing simple household chores to ease the burden on their parents.^31^ Those in the older age range felt a burden when they could not fulfill duties to provide for their family:

I just want to be more a man than I feel I am…want to be a rock, solid husband.^35^

Parents provided replacements or alternatives for gaps in their child’s life to create a sense of belonging.^22^ This advocacy continued across the continuum from adolescence into adulthood if they remained dependent, demonstrated by advocating support in school and also in the workplace when employment age was reached:

I had a word with them at the (social club)…And I asked the manager there if anything came up. (parent)^22^

Caregiving inevitably placed demands on the family unit, and in turn, parents acknowledged their own needs and how needs may change over time and sometimes decisions can become a source of distress:

It’s getting harder as he's become more of an adult, we've always had specific ideas about the ways things should be done and agreed up to this point.^21^

Furthermore, the needs of siblings are also a concern for the family:

It can be a strain and kind of push you…you gotta take care of your other kids too. (mom)^21^

Family functioning was reported in terms of family support provided to the survivor and the demands and strains this may place on the family unit; the greater the demand, the greater the stress on the family unit.

### Cancer-Related Factors That Challenge the Individual With a Brain Tumor

#### Living With Societal Expectations

Societal expectations place pressure on survivors to achieve typical milestones, for example, learning to drive, gaining academic qualifications, finding employment, dating, and having meaningful relationships.^5,22–25,30–33,39,40,46,47^ The nature of late effects and their severity can significantly influence the ability to achieve these goals. Disappointment was yet another burden for survivors to deal with when they just wanted to be perceived as “normal”; this need was present regardless of age^28,29,36^:

It’s constantly changing depending where you are in life and I take it like ok, we're good now. Then he turned 16 and wanted to drive, you're flying down the rollercoaster again and they you’re okay for a while, then he's 18 and wants to go away to college.^21^

Survivors reported carrying a “cancer” label and looked forward to opportunities where they may be able to lose this label, such as joining a new school or gaining employment where others were not aware of their history.^21^ Refutational analysis identified some survivors had a positive experience, whereas others found change did not always result in a feeling of being accepted:

Going to college was a release because it was a break from those people who knew about the cancer.^27^

Bullying was a recurring issue throughout the continuum of adolescence into adulthood whether at school or in the workplace and added to a sense of isolation^5,22–25,28,31,39,44,47^:

My classmates treated me as a toy, the often hit me for no reason and burned my arm with cigarettes.^31^

The hardest thing in my life is getting along with my friends…. I want to have friends but I can't seem to find them.^5^

Some parents and survivors discussed finding helpful support, describing the importance of a social program to lessen the feeling of isolation and promote a sense of belonging to a community; this was greeted with positivity^39^:

Every month he has something is a tremendous help for me. It is hard to find the right supportive social opportunity but at STEPS I have seen him be understood, people are patient, he is safe and has a good time.^39^

#### Barriers to Coping

Physical limitations such as impaired mobility as well as altered sight and speech caused by disease, treatment, or late effects pose a threat to reaching independence and can cause ongoing distress and influence the ability to adjust with positive coping styles. Refutational translation illustrated how time since diagnosis may influence these factors because some issues may only become apparent as time moves on and issues become a priority. For example, fertility may only impact the survivor once they reach the stage of wanting to start a family.^22,23,25^ Meaning the current age and stage of development is important when understanding the needs of survivors, not necessarily the time since treatment. The extent and severity of late effects caused by the disease and/or treatment impact the number of barriers to achieving independence. Some survivors may lack awareness of their own limitations, due to cognitive late effects, whereas others may be satisfied with remaining dependent on their parents. Ongoing dependency can cause further pressure on parents to educate and teach life skills, which some find difficult, as illustrated by the quote below:

I asked him the other day “Do you know what medicines you’re taking?” “Well, whatever you tell me to take.” And I tell him what type and how many times a day,…And he'll say, “That’s why I have you.”^40^

In addition to physical symptoms, caregivers and survivors feel ill-prepared to cope with late effects or where to seek help.^22,26,29,33,39,41,46^ Survivors and families reported not knowing how to get help at school because teachers have little information on how to support survivors of brain tumors. It often falls to parents to identify when problems arise in school, initiate extra support, and be the ones to share information on the needs of the young person, causing additional burden to the caregiver^5,20,22,28,46^:

Now there were no outward physical signs of being ill, her school didn’t think it was necessary to make accommodations for special education.^26^

Reciprocal analysis identified when adjustments were made, it provided a sense of relief:

The adjustments at work really make all the difference…so now I don’t have a lot of work tasks that I have to do simultaneously. I felt an immediate change when I got new tasks, I relaxed on a completely different level.^43^

Some parents found a constant lack of support and resources to help them cope with continual late effects associated with brain tumors. One caregiver reported problems with services designed to provide help, yet even they were ill-equipped to provide the support they needed:

I tried getting Ann help transitioning to adulthood from a non-profit group, she signed up for years. Finally they asked her to sign a release form saying she no longer needed their services, she complied. Truthfully, they did not know how to help someone with her cognitive disabilities. (parent)^26^

Many survivors and caregivers reported a lack of information about what to expect in the future or the potential late effects, and this inhibited their ability to prepare and manage the long-term sequelae:

Information on how things might evolve over time, and the long-term effects. It is sad to say it is absent but it’s close to the truth.^22^

The constant and unpredictable nature of late effects was summed up as:

The experience is a lifetime deal, continually wonder when things will get easier, there’s no light at the end of the tunnel.^21^

Survivors may experience multiple ongoing barriers due to the late effects of their disease and treatment. In addition to this, the pressure to meet social expectations adds another burden to deal with, exacerbating negative emotions and requiring resilience to manage ongoing issues. Studies report a lack of effective information and resources to help survivors and their families manage these issues.^22,27,38,42,46^

I don’t think you get as much support as you should do…the further away your appointments get…you still come up against a lot of problems and there isn’t really anybody there.^22^

Where support has been identified and used, in the school setting and peer groups, this was met with great relief.

### Line of Argument: A Conceptual Framework of the Resilience of Survivors and Ongoing Challenges

**Figure 2:**
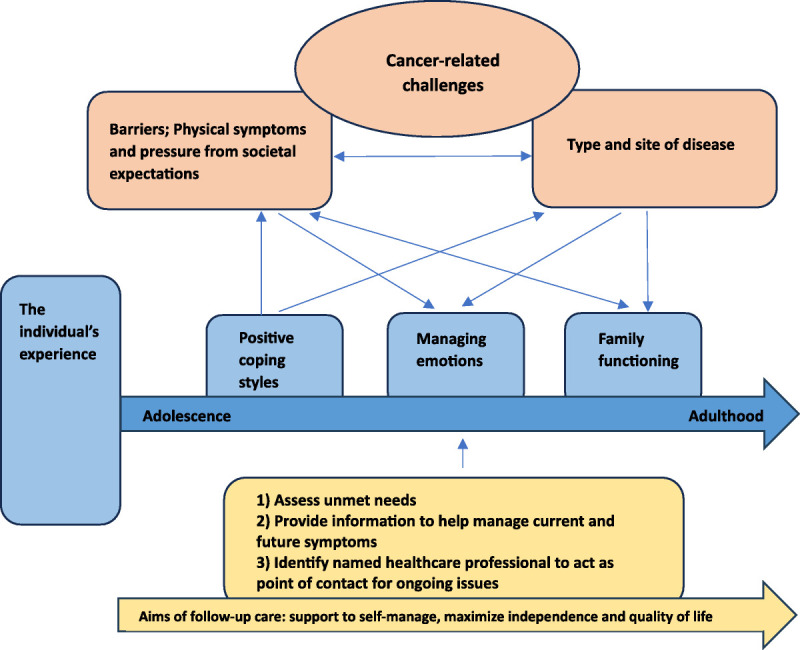
Conceptual framework: a model to represent the experiences of adolescent and young adult survivors of a brain tumor and the role of follow-up care.

The process of synthesis, using reciprocal and refutational translation, enabled us to generate a conceptual framework (Figure 2) illustrating the interrelationship of themes representing characteristics and perceptions expressed by survivors, which are integral to individuals regardless of their place on the continuum of adolescence into adulthood. The relationships between themes were considered in combination with the support advocated from follow-up services to produce the final framework.

The conceptual framework reflects the experiences at the attained age, between adolescence and adulthood, addressing our first objective to examine time since diagnosis. This age range is a time of rapid growth with key milestones; it highlights the vulnerability of this group to potential disappointment and the need for support to manage expectations and respond when milestones cannot be reached because of ongoing adverse effects of treatment. Current information suggests the family carry the responsibility for overcoming the impact of physical barriers and lessening the disappointment when societal expectations cannot be achieved. By incorporating parental perceptions in this review, we were able to demonstrate how families create opportunities to enable the individual to develop and realize their potential. It also allowed us to understand where families have a greater role for those whose treatment and diagnosis were more severe.

Our objectives were achieved when added details regarding the individual’s age, diagnosis, and treatment were reported with first-order data, allowing us to understand the individual experiences in relation to their age and disease type. All themes related to individuals at any age between 15 and 39 years. The type of disease influenced the presence of negative emotions, family functioning, and pressures from societal expectations. Refutational synthesis illustrated the ability to exhibit positive emotions, which remained across the age groups and regardless of disease type, presence, or intensity of physical barriers. In addition, support and information to manage new or ongoing symptoms was often lacking, although where it was provided, it was met with great relief and comfort. For many with ongoing symptoms, as time from treatment increased, survivors and families felt less support was available, and furthermore, they did not know who to ask for ongoing support.

## Discussion

This review synthesizes qualitative data exploring the needs and experiences of survivors of a brain tumor aged between 15 and 39 years, including parental perceptions.

Improving quality of life, aiding a return to normalcy, and supporting individuals to reach milestones and gain independence are the ambition of individuals, families, and follow-up services. However, as witnessed in this review, gaining independence hinges on the presence of late effects from disease and treatment. Reciprocal and refutational analysis demonstrated those with severe late effects associated with the type and site of disease/treatment suffered more from physical barriers causing distress when societal demands and significant milestones cannot be reached, causing more challenging emotions to be experienced. Table 4 illustrates reciprocal and refutational analysis across the themes. However, if the ability to remain positive is synonymous with low levels of unmet needs and perceived HRQOL,^3,48,49^ then this review refutes existing knowledge, which reports a positive attitude was present despite high levels of unmet needs. Previous studies also report ambiguity around measures of “quality of life,” demonstrated by Beecham and colleagues^12^ who reported that an emphasis on health status may neglect other aspects of life important to individuals’ experience of survivorship and measuring HRQOL may not be representative of unmet needs. Quantitative studies also report discrepancies when measuring HRQOL,^50,51^ implying that an HRQOL assessment may overlook individuals’ needs. Clinical implications of this indicate an individual assessment of need may be necessary to provide individualized support in follow-up. Despite the development of age-appropriate needs assessment tools, these are not disease specific and may not fully address the needs of survivors of a brain tumor.^52,53^ Similarly, currently available brain tumor-specific needs assessment tools for adults may not be suitable to detect the needs of AYAs.^9,48,52^

**Table 4:** Examples of Refutational Analysis: Quotes of Reciprocal and Refutational Translation for Each Theme

Themes	Reciprocal Translation	Refutational Translation
Positive coping styles	“If you want to get better quickly you have to have a positive attitude…. You just need to be positive…and just do things that you normally are going to do because if you don’t, then you’re not going to be called a survivor.”^36^	“I felt guilty for the credit of being called survivor. And I don’t want my future life to be associated with sadness, anxiety and stress of cancer.”^24^
Managing emotions	“We don’t focus on the condition, it used to be the 1st thing, that is not how life works, life is hard for all kids at one point.”^21^ “To me it means just—living your life to the most—doing whatever you can to enjoy life and use that second chance you’ve been given.”^36^	“We’re all scared man.”^28^ “It scares the living daylights out of me, the thought of it coming back and having to go through that again.”^35^
Family functioning	“I just want to be more a man than I feel I am. I want to be a rock, a support, solid husband…. I want to go and insulate the loft, I want to be a reliable husband.”^30^ “It’s getting harder as he’s become more of an adult, we’ve always had specific ideas about the ways things should be done and agreed up to this point.”^21^	“We just roll with the punches, you know, his normalcy is his normalcy, I don’t see differences.”^21^
Living with societal expectations	“Going to college was a release because it was a break from those people who knew about the cancer.”^27^	“I can’t drive and I can’t do all the things that other people my age are doing.”^39^
Barriers to coping	“The adjustments at work really make all the difference…so now I don’t have a lot of work tasks that I have to do simultaneously. I felt an immediate change when I got new tasks, I relaxed on a completely different level.”^43^	“She went through a tremendous amount of bullying, so that’s why I decided to teach them at home.”^22^ “Nobody wants to know your name when they think you’re stupid.”^28^

This review has highlighted how individuals feeling in control of their emotions and physical symptoms and those able to positively help themselves portrayed a greater satisfaction of self and family functioning; therefore, opportunities to improve the ability for individuals to self-manage symptoms must be taken, and late effects services are in an ideal position to facilitate this. Support to self-manage symptoms can empower individuals and possibly minimize late effects. This review suggests survivors are not always aware of information required to self-manage symptoms or make informed decisions regarding their lifestyle choices, and this lack of awareness of late effects can hinder an individual’s ability to manage them. Furthermore, it is well known that young adults display feelings of being “invincible,” demonstrated by the survivor “feeling like Superman”; this attitude may influence their perceived susceptibility and threat of late effects. Ongoing support must acknowledge the nature of young adults and their decision-making ability when navigating the steps to manage their own health. In addition, cognitive impairment may further limit the ability for an individual to process information to self-manage symptoms. Decision-making ability and cognitive impairment are important to consider when providing information, which is highlighted as a key aspect of support.^24,40,43^

This review reinforces the need to acknowledge the family’s role in managing late effects. Caregiver needs and the family’s ability to access resources when supporting the individual regardless of age are fundamental to the life of a survivor and their potential for managing late effects. Consideration of their needs is even more necessary for those who have undergone more intense treatment and continue with intense late effects.

## Strengths and Limitations

The concept that the quality of qualitative studies lies in the quality of the metaphor directed the approach to quality appraisal.^14^ Therefore, a decision regarding the quality of the study was applied by judging the study’s ability to produce new theory by going beyond a thematic analysis. Some may criticize the lack of a more formal checklist; however, this method was supported by Dixon-Woods et al,^53^ because measuring qualitative studies against a fixed set of criteria produces the same level of agreement as a reviewer’s unprompted, expert judgment. Studies of all quality were included, and we acknowledge this approach potentially allows the inclusion of data from studies where the researcher may have influenced data collection.

Our meta-ethnography is representative of the experiences of survivors of brain tumors as expressed and reported in the included studies. It is worth noting that, despite including populations with any ethnic, cultural, or religious background, we almost solely found studies in White populations. This means our findings did not represent other ethnic groups.

## Conclusion

The conceptual framework illustrates the challenges survivors experience alongside aspects of support that could be provided by the follow-up services regarding provision of information and assessing needs. Routine assessment of unmet needs could identify individuals and families that need and want support while also informing personalized follow-up, particularly to relieve the pressure on those families who become condition focused.^20,38^

## Implications for Practice

In addition to routine assessment of needs, a nominated contact at the treating center would mean those who have issues know where to access support and improve access to information. This support should include opportunities to harness resilience and enable individuals to self-manage symptoms.^41^ Finally, follow-up services should consider adopting interventions that have been proven to lessen social isolation, such as the Success Through Education, Psychosocial Support and Socialization Program.^39^ Interventions may also include opportunities to provide advocacy in education and employment. Further research is warranted to develop and evaluate interventions to overcome barriers to reintegration into society, a process that this review highlighted as relentless for some.

## Supplementary Material

SUPPLEMENTARY MATERIAL
