# Supplementary material for: Experiences and Unmet Needs of Adolescent and Young Adult Survivors of a Brain Tumor (Aged 15–39 Years): A Systematic Review and Meta-ethnography
Source: Cancer Nurs. 2023 Dec 8;48(4):307–15. doi: 10.1097/NCC.0000000000001311 (PMC12237130; doi:10.1097/NCC.0000000000001311)
Supplement: SUPPLEMENTARY MATERIAL [file cn-48-0307-s001.docx]

**Introduction**

Brain and central nervous system tumors are the most common solid tumor across 153 cancer registries worldwide for those aged 0-19 years.^1^ They account for 15% of adolescent and young adult (AYA) cancers aged 15-25 years.^2^ Adolescent and young adults aged 15 to 39 have the highest rates of survival following a brain tumor, which when combined with the incidence rate in this age group, indicates this population has the largest proportion of survivors predisposed to late effects. More than half of survivors of a brain tumor have ongoing symptoms that can have a detrimental impact on an individual’s ability to attain common milestones reached during adolescence.^3^ Failure to achieve these milestones highlights the susceptible nature of adolescence and the vulnerability of those severely affected by their diagnosis and treatment. Lower rates of marriage and employment, social isolation and limits on reaching independence are a consequence of late effects from disease and treatment.^3-6^

The aim of support for cancer survivors is to maximize quality of life by aiding a return to normality and independence as soon as possible while minimizing the negative effects associated with cancer and its treatment.^7^ Recommendations for follow-up services advocate and incentivize implementation of personalized packages of follow-up care comprising of: well-being events aimed to support self-management of symptoms; rapid access to a cancer center when necessary; completion of holistic needs assessments; and access to health and well-being information.^8^ In addition, survivors of a brain tumor should be made aware that lifestyle choices may impact on the severity and management of late effects. Therefore, follow-up services need to acknowledge typical behavior traits displayed in this age group: immature decision-making and not placing emphasis on the long-term consequences of decisions.^9^ A better understanding of all aspects of the survivorship experience of AYAs is required to ensure follow-up services are able to meet the needs of this population.

Systematic reviews examining quality of life and experiences of brain tumor survivors mainly focus on adult^10,11^ or pediatric populations.^12,13^ This meta-ethnography aims to add to existing knowledge by aggregating qualitative data and interpretively synthesizing this to generate new concepts regarding all aspects of the survivorship experience. In order to identify areas of unmet needs for young people aged 15 to 39 years, the focus of this review is on quality of life, family functioning, experiences of physical and cognitive late effects and social functioning, including the perspectives of parents, partners and caregivers.

**Objectives**

Primary:

To identify and synthesize qualitative studies to better understand the experiences, perspectives and needs of AYA brain tumor survivors (aged 15 to 39 years).

Secondary:

1. Explore the effect of time since diagnosis on the needs of young people as they progress through adolescence.
2. Examine how the type of brain tumor and/or treatment may impact the experience and needs of survivors.
3. Examine parental perceptions of the young persons’ needs.

**Methods**

A systematic search strategy and meta-ethnography guided by the seven phases outlined by Noblit and Hare^14^ was conducted and the report follows France et al’s^15^ guidance to maximize transparency. Noblit and Hare^14^ suggested the following seven steps: getting started; deciding what is relevant; reading the studies; determining how studies are related; translating studies; synthesizing translation; and expressing the synthesis. The research team consisted of clinicians and researchers with expertise in Adolescent and Young Adult oncology and meta-ethnography. Conducting a systematic review did not require ethical board approval.

**Search strategy and screening process**

A pre-planned electronic search of published articles written in the English language between 2000-2021 was conducted using the PEO framework^16^ to identify search concepts related to survivors (population), brain tumors (exposure), and experience (outcomes) (see the Appendix, http://links.lww.com/CN/A177 for an example of search history in MEDLINE). The timeline of the search strategy reflects the establishment of AYA cancer services in the United Kingdom and internationally, as well as the implementation of national strategies to ensure provision of age-appropriate services for all young people.^17^ Databases from nursing, medical and social science disciplines were searched to maximize the capture of the experience of survivors from all perspectives. We also hand-searched reference lists, Google Scholar and specific prominent journals where information on survivorship is largely published (such as Journal of Adolescent and Young Adult Oncology, Journal of Pediatric Hematology/ Oncology Nursing and Psycho-Oncology).

**Inclusion and Exclusion criteria**

Two independent researchers (KL and IS) completed two-stage screening of titles and abstracts of the retrieved studies according to the inclusion/ exclusion criteria (Table 1).

The first stage screening excluded those not meeting the inclusion criteria. Full text screening was carried out in stage two and recording the reason for exclusion. Disagreements were resolved through discussion with a third member of the team (FG).

Where studies included populations with mixed cancer types, studies were included if quotes from survivors of a brain tumor could be identified and extracted. Similarly, where the study population range was outside of 15 to 39 years, studies were included if quotes of participants within our defined age range were identifiable. This allowed an inclusive approach to ensuring all studies reporting qualitative data regarding the experiences of AYA survivors of a brain tumor aged 15 to 39 years could be included.

**Data extraction**

Study characteristics were documented in a summary data table to enable an understanding and comparison of the data. Contextual information such as the diagnosis and type of treatment were recorded with the participants’ quotes where available to allow exploration of the secondary objectives. In congruence with meta-ethnography,^18^ studies were not excluded due to quality or ability to produce rich data (that which reveals the complexities of what is being studied).^14^ Quality assessment was carried out by two independent researchers (KL and IS) and judged the ability of a study to produce higher level analysis that goes beyond a narrative. Criteria by Walsh and Downe^19^ was used to make an objective decision regarding quality (Yes, No, Unclear) and is reported in the summary data table with examples to support decisions made.

**Data synthesis**

Data synthesis involves systematically comparing studies to identify similarities and differences between the included studies, known as reciprocal and refutational translation respectively. Quotes, known as first order data, from each study can be used as evidence to support reciprocal translation between studies, where similarities exist.^14^ Where quotes oppose the reciprocal translation, or report contradictory information, this is an example of refutational translation. Data synthesis consists of the construction of themes (third order data) that incorporate reciprocal and refutational translation. For this meta-ethnography, we analyzed first order data and second order data, and the original authors concepts together rather than in isolation to generate the new line of argument and conceptual framework.^17^ Codes were developed to label first order data which were taken from the results section of each paper, during an iterative process of reading, re-reading and interpretation to allow the researchers to determine how studies were related. Codes were then tabulated to allow data to be compared and contrasted, allowing the translation phase of synthesis (Table 2). Similar codes were grouped into concepts. Two researchers (KL and IS) completed this stage to minimize bias when allocating codes to data.

Multiple discussions between three members of the research team (FG, KL and IS) allowed themes to be developed through synthesizing and identifying reciprocal or refutational codes. The final stage of analysis was to draw conceptual maps illustrating relationships between themes and demonstrate synthesizing translations. Discussion and translation of themes enabled us to use our third order data to develop a new line of argument, forming a conceptual framework regarding the ongoing daily experience of surviving a brain tumor. The framework was developed through five iterations of the concept map.

**Results**

Figure 1 highlights the search and screening process. Study characteristics and themes were documented and included in the synthesis.

**Study Characteristics**

Twenty-nine studies from seven different countries met the inclusion criteria. Table 3 summarizes the study characteristics, main findings and critical appraisal.

Two studies included mixed cancer types where quotes from survivors of a brain tumor could be identified.^24,25^ The review included studies representing different life experiences of survivors such as family functioning, cognitive late effects, health-related quality of life (HRQOL) and education or employment. One paper was a personal narrative of one parent’s experience of caring for a childhood survivor of a brain tumor^26^ and two papers were reflections from a nurse’s experience of joining a peer support group attended by survivors of brain tumors.^27,28^

**Themes**

Reciprocal and refutational translation of studies revealed concepts relating to the individuals’ experience of life following treatment for a brain tumor, which were represented across the continuum from adolescence to adulthood. We were able to address our objectives and each theme incorporates the effect of time since diagnosis, the impact of different types of tumor and treatment on these experiences and parental perceptions where possible. Two themes and five sub-themes are reported and represent aspects of survivors’ lives. The first theme related to the individual and sub-themes were seen as integral to the daily life of survivors of a brain tumor. The second theme related to the impact of cancer, treatment and its late effects on the person.

1. **Individual factors impacting the resilience of survivors**

**Positive coping styles**

The individual nature of coping styles was apparent from reciprocal translation of all included studies regardless of age, diagnosis or severity of late effects and many survivors and caregivers were able to find meaning and positivity from their experience, portraying a strong determination and drive to achieve their goals:^23,28-29,32^

“*the doctors tell me I would never graduate high school…and I was like, yeah, I'm showing you.. I was able to do it*”.^29^

Other positive attitudes continued to emerge throughout studies regardless of the age or diagnosis of the survivor. Where positive ambition and ability to adjust exist, young people were able to maintain some control over their situation.^20,21,27,29,31,36,40,43^

Positive attitudes were evident by young people’s reflections on their situation:

“*after everything I've been through, I feel like superman*”.^20^

The concept of feeling like a survivor had both positive and negative implications, demonstrating refutational analysis within this theme. Some felt they did not deserve the title of survivor, while others felt the term survivor labeled a traumatic event and kept the diagnosis present. In addition to the internal drivers of positive coping, having a faith contributed to making sense and maintaining control of the situation:

“*God picked me for a reason. He picked me to teach him how to deal with this*”.^34^

Individual coping styles were apparent throughout the studies, representing individual resilience and were reported by participants regardless of the severity of disease/treatment or age. However, the ability to remain positive was conflicted with the presence of different emotions expressed by individuals.

**Managing emotions**

Despite the ability to maintain positivity, reciprocal translation of studies also found survivors of all ages portraying a range of negative emotions such as persistent anxiety, depression and suicidal thoughts, adding a burden on survivors and their families.^22,23,29,31,40^

“*I didn't want to tell my parents I was injured by my classmates, so I banged the wall to hurt my hands in my bedroom and considered suicide..*”^31^

Feelings of anger and frustration spanned the types of disease and treatment, though they were felt to a greater extent in those whose late effects were more limiting. Feelings of inadequacy and poor self-esteem due to perceiving themselves as a burden on the family were also reported:

“*sometimes you feel like your value to your family or society is not as good as the person next to you. I'm not making any contribution to society by going there and working. You feel less of a person*”.^23^

A sense of loss was common to survivors: loss of the life planned/old life, loss of friendships and loss of physical function that impacted on a survivor’s day to day*.*^22,23,26,38,39,46^ Many survivors and caregivers were scared of the unpredictable nature of cancer and described fear of recurrence or fear for their future.^22,27,35,38,39,41,43,46^

“I *don't ever relax.. If you get too comfortable that’s when things will happen*”.^41^

It is clear from the literature that survivors continue to experience a lasting range of emotions that can help or hinder their day-to-day life and persistent late effects influence the number of negative emotions being expressed.

**Family functioning**

The significance of support from family members from adolescence into adulthood was acknowledged as being crucial throughout this continuum and studied by many.^20-22,41,44-46^ Refutational analysis identified the change in the definition of family depending on the individual's place along the continuum from adolescence into adulthood. Significant others were parents and siblings at the younger end of this spectrum while partners/spouse took this place for older individuals. Families who are able to remain “family-focused” have a greater ability to adapt, incorporate management of any conditions into family life and report higher HRQOL compared with families who were “condition-focused”.^20,44^ The ability to remain “family-focused” was directly related to treatment intensity and where families were condition-focussed, the survivor was more likely to have experienced intense treatment and severe late effects. Where the impacts are ongoing, there is a constant feeling of anticipation^21,44-47^:

*“I’d like to say that her condition is not the most important thing in our family, but there’s not a day that goes by that I don’t think about it. Either I am thinking about the late effects or the chance of it coming back. It’s like waiting for the other shoe to fall*”.^21^

Family functioning was also described by Hammond^26^ and Lucas et al^40^ who demonstrated higher levels of anxiety and uncertainty were associated with increased caregiver demands. Where survivors had a greater dependence on their family, they also relied on them for their main source of social support^47^:

“*my mother in particular is my backbone*”. ^20^

Survivors felt a burden on their family across the continuum of adolescence into adulthood. Younger adolescents recognized how much their parents did for them and wanted to return their help by doing simple household chores to ease the burden on their parents.^31^ Those in the older age range felt a burden when they could not fulfill duties to provide for their family:

“*I just want to be more a man than I feel I am.. want to be a rock, solid husband..*”.^35^

Parents provided replacements or alternatives for gaps in their child’s life to create a sense of belonging.^22^ This advocacy continued across the continuum from adolescence into adulthood if they remained dependent, demonstrated by advocating support in school and also in the workplace when employment age was reached:

“*I had a word with them at the (social club).. And I asked the manager there if anything came up. (parent)”*.^22^

Caregiving inevitably placed demands on the family unit and in turn parents acknowledged their own needs and how needs may change over time and sometimes decisions can become a source of distress:

“*it’s getting harder as he's become more of an adult, we've always had specific ideas about the ways things should be done and agreed up to this point*”.^21^

Furthermore, the needs of siblings are also a concern for the family:

“*it can be a strain and kind of push you… you gotta take care of your other kids too.. (mum*)”.^21^

Family functioning was reported in terms of family support provided to the survivor and the demands and strains this may place on the family unit, the greater the demand, the greater the stress on the family unit.

1. **Cancer-related factors that challenge the individual with a brain tumor:**

**Living with societal expectations**

Societal expectations place pressure on survivors to achieve typical milestones, for example, learning to drive, gaining academic qualifications, finding employment, dating and having meaningful relationships.^5,22-25,30-33,39-40,46-47^ The nature of late effects and their severity can significantly influence the ability to achieve these goals. Disappointment was yet another burden for survivors to deal with when they just wanted to be perceived as “normal”; this need was present regardless of age:^28-29,36^

“*it’s constantly changing depending where you are in life and I take it like ok, we're good now. Then he turned 16 and wanted to drive, you're flying down the rollercoaster again and they you’re okay for a while, then he's 18 and wants to go away to college*”.^21^

Survivors reported carrying a “cancer” label and looked forward to opportunities where they may be able to lose this label, such as joining a new school or gaining employment where others were not aware of their history.^21^ Refutational analysis identified some survivors had a positive experience while others found change did not always result in a feeling of being accepted:

“*going to college was a release because it was a break from those people who knew about the cancer*”.^27^

Bullying was a recurring issue throughout the continuum of adolescence into adulthood whether at school or in the workplace and added to a sense of isolation:^5,22-25,28,31,39,44,47^

“*my classmates treated me as a toy, the often hit me for no reason and burned my arm with cigarettes*”.^31^

And “*the hardest thing in my life is getting along with my friends.. I want to have friends but I can't seem to find them*”.^5^

Some parents and survivors discussed finding helpful support, describing the importance of a social program to lessen the feeling of isolation and promote a sense of belonging to a community; this was greeted with positivity:^39^

“*every month he has something is a tremendous help for me. It is hard to find the right supportive social opportunity but at STEPS I have seen him be understood, people are patient, he is safe and has a good time*”.^39^

**Barriers to coping**

Physical limitations such as impaired mobility, altered sight and speech caused by disease, treatment or late effects pose a threat to reaching independence and can cause ongoing distress and influence the ability to adjust with positive coping styles. Refutational translation illustrated how time since diagnosis may influence these factors since some issues may only become apparent as time moves on and issues become a priority. For example, fertility may only impact the survivor once they reach the stage of wanting to start a family.^22,23,25^ Meaning the current age and stage of development is important when understanding the needs of survivors, not necessarily the time since treatment. The extent and severity of late effects caused by the disease and/or treatment impacts the number of barriers to achieving independence. Some survivors may lack awareness of their own limitations, due to cognitive late effects, while others may be satisfied with remaining dependent on their parents. Ongoing dependency can cause further pressure on parents to educate and teach life skills, which some find difficult, illustrated by the quote below:

“*I asked him the other day "do you know what medicines you're taking?" "well, whatever you tell me to take". And I tell him what type and how many times a day, .. And he'll say, "that’s why I have you*".^40^

In addition to physical symptoms, caregivers and survivors feel ill-prepared to cope with late effects or where to seek help.^22,26,29,33,39,41,46^ Survivors and families reported not knowing how to get help at school as teachers have little information on how to support survivors of brain tumors. It often falls to parents to identify when problems arise in school, to initiate extra support and be the ones to share information on the needs of the young person, causing additional burden to the caregiver:^5,20,22,28,46^

“*Now there were no outward physical signs of being ill, her school didn't think it was necessary to make accommodations for special education*”.^26^

Reciprocal analysis identified when adjustments were made, it provided a sense of relief:

“*the adjustments at work really make all the difference… so now I don't have a lot of work tasks that I have to do simultaneously. I felt an immediate change when I got new tasks, I relaxed on a completely different level*”.^43^

Some parents found a constant lack of support and resources to help them cope with continual late effects associated with brain tumors. One caregiver reported problems with services designed to provide help yet even they were ill-equipped to provide the support they needed:

“*I tried getting Ann help transitioning to adulthood from a non-profit group, she signed up for years. Finally they asked her to sign a release form saying she no longer needed their services, she complied. Truthfully, they did not know how to help someone with her cognitive disabilities. (parent*)”.^26^

Many survivors and caregivers reported a lack of information about what to expect in the future or the potential late effects and this inhibited their ability to prepare and manage the long-term sequelae:

“*Information on how things might evolve over time, and the long-term effects. It is sad to say it is absent but it’s close to the truth*”.^22^

The constant and unpredictable nature of late effects was summed up as:

“*the experience is a lifetime deal, continually wonder when things will get easier, there’s no light at the end of the tunnel*”.^21^

Survivors may experience multiple ongoing barriers due to the late effects of their disease and treatment. In addition to this, the pressure to meet social expectations adds another burden to deal with, exacerbating negative emotions and requiring resilience to manage ongoing issues. Studies report a lack of effective information and resources to help survivors and their families manage these issues.^22,27,38,42,46^

*“I don’t think you get as much support as you should do… the further away your appointments get.. you still come up against a lot of problems and there isn’t really anybody there*^”22^

Where support has been identified and utilized, in the school setting and peer groups, this was met with great relief.

**Line of Argument: A Conceptual Framework of the resilience of survivors and ongoing challenges**

The process of synthesis, using reciprocal and refutational translation enabled us to generate a conceptual framework (Figure 2) illustrating the inter-relationship of themes representing characteristics and perceptions expressed by survivors which are integral to individuals regardless of their place on the continuum of adolescent into adulthood. The relationships between themes were considered in combination with the support advocated from follow-up services to produce the final framework.

The conceptual framework reflects the experiences at the attained age, between adolescence and adulthood, addressing our first objective to examine time since diagnosis. This age range is a time of rapid growth with key milestones; it highlights the vulnerability of this group to potential disappointment and the need for support to manage expectations and respond when milestones cannot be reached due to ongoing side effects of treatment. Current information suggests the family carry the responsibility for overcoming the impact of physical barriers and lessening the disappointment when societal expectations cannot be achieved. By incorporating parental perceptions in this review, we were able to demonstrate how families create opportunities to enable the individual to develop and realize their potential. It also allowed us to understand where families have a greater role for those whose treatment and diagnosis were more severe.

Our objectives were achieved when added details regarding the individual’s age, diagnosis and treatment were reported with first order data allowing us to understand the individual experiences in relation to their age and disease type. All themes related to individuals at any age between 15 and 39 years. The type of disease influenced the presence of negative emotions, family functioning and pressures from societal expectations. Refutational synthesis illustrated the ability to exhibit positive emotions which remained across the age groups and regardless of disease type, presence or intensity of physical barriers. In addition, support and information to manage new or ongoing symptoms was often lacking, though where it was provided, it was met with great relief and comfort. For many with ongoing symptoms, as time from treatment increased, survivors and families felt less support was available and furthermore, they did not know who to ask for ongoing support.

**Discussion**

This review synthesizes qualitative data exploring the needs and experiences of survivors of a brain tumor aged between 15 and 39 years, including parental perceptions.

Improving quality of life, aiding a return to normalcy, and supporting individuals to reach milestones and gain independence are the ambition of individuals, families and follow-up services. However, as witnessed in this review, gaining independence hinges on the presence of late effects from disease and treatment. Reciprocal and refutational analysis demonstrated those with severe late effects associated with the type and site of disease/treatment, suffered more from physical barriers causing distress when societal demands and significant milestones cannot be reached, causing more challenging emotions to be experienced. Table 4 illustrates reciprocal and refutational analysis across the themes. However, if the ability to remain positive is synonymous with low levels of unmet needs and perceived HRQOL,^3,48-49^ then this review refutes existing knowledge which reports a positive attitude was present despite high levels of unmet needs. Previous studies also report ambiguity around measures of “quality of life”, demonstrated by Beecham and colleagues^12^ who reported that an emphasis on health status may neglect other aspects of life important to individuals' experience of survivorship and measuring HRQOL may not be representative of unmet needs. Quantitative studies also report discrepancies when measuring HRQOL,^50-51^ implying that a HRQOL assessment may overlook individuals’ needs. Clinical implications of this indicate an individual assessment of need may be necessary to provide individualized support in follow-up. Despite the development of age-appropriate needs assessment tools, these are not disease-specific and may not fully address the needs of survivors of a brain tumor.^52,53^ Similarly, currently available brain tumor-specific needs assessment tools for adults may not be suitable to detect the needs of AYAs.^9,48,52^

This review has highlighted how individuals feeling in control of their emotions and physical symptoms and those able to positively help themselves portrayed a greater satisfaction of self and family functioning; therefore, opportunities to improve the ability for individuals to self-manage symptoms must be taken and late effects services are in an ideal position to facilitate this. Support to self-manage symptoms can empower individuals and possibly minimize late effects. This review suggests survivors are not always aware of information required to self-manage symptoms or make informed decisions regarding their lifestyle choices and this lack of awareness of late effects can hinder an individual’s ability to manage them. Furthermore, it is well known that young adults display feelings of being “invincible”, demonstrated by the survivor “feeling like Superman”; this attitude may influence their perceived susceptibility and threat of late effects. Ongoing support must acknowledge the nature of young adults and their decision-making ability when navigating the steps to manage their own health. In addition, cognitive impairment may further limit the ability for an individual to process information in order to self-manage symptoms. Decision-making ability and cognitive impairment is important to consider when providing information which is highlighted as a key aspect of support.^24,40,43^

This review reinforces the need to acknowledge the family's role in managing late effects. Caregiver needs and the family’s ability to access resources when supporting the individual regardless of age is fundamental to the life of a survivor and their potential for managing late effects. Consideration of their needs is even more necessary for those who have undergone more intense treatment and continue with intense late effects.

**Strengths and Limitations**

The concept that the quality of qualitative studies lies in the quality of the metaphor directed the approach to quality appraisal.^14^ Therefore, a decision regarding the quality of the study was applied by judging the study's ability to produce new theory by going beyond a thematic analysis. Some may criticize the lack of a more formal checklist; however, this method was supported by Dixon-Woods et al^53^ since measuring qualitative studies against a fixed set of criteria produces the same level of agreement as a reviewer’s unprompted, expert judgment. Studies of all quality were included, and we acknowledge this approach potentially allows the inclusion of data from studies where the researcher may have influenced data collection.
Our meta-ethnography is representative of the experiences of survivors of brain tumors as expressed and reported in the included studies. It is worth noting that, despite including populations with any ethnic, cultural or religious background, we almost solely found studies in Caucasian populations. This means our findings did not represent other ethnic groups.

**Conclusion**

The conceptual framework illustrates the challenges survivors experience alongside aspects of support that could be provided by the follow-up services regarding provision of information and assessing needs. Routine assessment of unmet needs could identify individuals and families that need and want support while also informing personalized follow-up, particularly to relieve the pressure on those families who become condition-focused.^20,38^

**Implications for practice**

In addition to routine assessment of needs, a nominated contact at the treating center would mean those who have issues know where to access support and improve access to information. This support should include opportunities to harness resilience and enable individuals to self-manage symptoms.^41^ Lastly, follow-up services should consider adopting interventions that have been proven to lessen social isolation, such as the Success Through Education, Psychosocial support and Socialization program.^37^ Interventions may also include opportunities to provide advocacy in education and employment. Further research is warranted to develop and evaluate interventions to overcome barriers to reintegration into society, a process that this review highlighted as relentless for some.

**References**

1. Steliarova-Foucher E, Colomet M, Ries LAG, et al. International incidence of childhood cancer 2001-10: a population-based registry study. *Lancet Oncology.* 2017;18(6):719-731.

2.<http://www.ncin.org.uk/cancer_type_and_topic_specific_work/cancer_type_specific_work/cancer_in_children_teenagers_and_young_adults/>. Accessed February 22, 2022.

3. Bhat SR, Goodwin TL, Burwinkle TM, et al. Profile of Daily Life in Children with Brain Tumours: An Assessment of Health Related Quality of Life. *J of Clin Oncol.* 2005;3(24):5493-5500.

4. Frobisher C, Lancashire ER, Winter DL, Jenkinson DL, Jenkinson HC. Long-Term Population Based Marriage Rates Among Adult Survivors of Childhood Cancer in Britain. *Int J of Cancer.* 2007;121(4):846-855.

5. Hobbie WL, Ogle S, Reilly M, et al. Adolescent and young adult survivors of childhood brain tumours. *Cancer Nurs.* 2016;39(2):134-144

6. Khan F, Amatya B, Drummond K, Galea M. Effectiveness of Integrated Multidisciplinary Rehabilitation in Primary Brain Cancer Survivors in an Australian Community Cohort: A Controlled Clinical Trial. *J of Rehab.* 2014;46(8):754-760.

7. Glaser A, Levitt G, Morris P, Tapp J, Gibson F. Enhanced Quality and Productivity of Long-Term Aftercare of Cancer in Young People. *Arch of Disease in Childhood.* 2013;98(10):818-824

8. Independent Taskforce for Cancer. Achieving world class cancer outcomes. A strategy for England 2015-2020. 2015.

9. Wakefield CE, Patterson P, McDonald FEJ, Wilson HL, Davis EL. Assessment of Psychosocial Outcomes in Adolescents and Young Adults with Cancer: A Systematic Review of Available Instruments. *J of Clin Oncol in Adolesc and Young Adults.* 2013;3:13-27.

10. Afseth J, Neubeck L, Karatzias T, Grant R. Holistic Needs Assessment in Brain Cancer Patients: A Systematic Review of Available Tools. *Eur J Cancer Care.* 2019;28(3):e12931.

11. Cubis L, Ownsworth T, Pinkham MB, et al. The social trajectory of brain tumor: a qualitative meta-synthesis. *J of Disability and rehabilitation.* 2018;40(16):1857-1869.

12. Beecham E, Langner R, Hargrave D, Bluebond-Langner M. Children’s and Parent’s Conceptualisation of Quality of Life in Children with Brain Tumours: A Meta-Ethnographic Exploration. *Qual Health Res.* 2019;29(1):55-68.

13. Woodgate RL, Tailor K, Yanofsky R, et al. Childhood brain cancer and its psychosocial impact on survivors and their parents: a qualitative thematic synthesis. *Eur J of Oncol Nurs.* 2016;20:140-149.

14. Noblit GW, Hare RD. Meta-ethnography. *Synthesizing qualitative studies*. SAGE publishing. 1988.

15. France EF, Cunningham M, Ring N, et al. Improving Reporting of Meta-Ethnography: The eMERGe Reporting Guidance. *BMC Medical Res Method* 2019;19(1):25.

16. Bettany-Saltikov J, McSherry R. How to do a Systematic Literature Review in Nursing: A step-by-step guide (2nd Edition). 2016.

17. Department of Health. The NHS Cancer Plan. A plan for investment. A plan for reform. [www.nhs.uk/nhsplan 2000](http://www.nhs.uk/nhsplan%202000). Accessed March 23, 2022.

18. France EF, Uny I, Ring N, et al. A Methodological Systematic Review of Meta-Ethnography Conduct to Articulate the Complex Analytical Phases. *BMC Res Method.* 2019b;19(1):35.

19. Walsh D, Downe S. Appraising the quality of qualitative research. *Midwifery*. 2006;22(2):108-119.

20. Hocking MC, Hobbie WL, Deatrick JA, et al. Neurocognitive and Family Functioning and Quality of Life Among Young Adult Survivors of Childhood Brain Tumors. *The Clinical Neuropsychologist.* 2011;25(6):942-962.

21. Deatrick JA, Mullaney EK, Mooney-Doyle K. Exploring Family Management of Childhood Brain Tumor Survivors. *J of Ped Oncol Nurs.* 2009;26(5):303-311.

22. Vance YH, Eiser C, Horn B. Parents Views of the Impact of Childhood Brain Tumours and Treatment on Young Peoples Social and Family Functioning. *Clinical Child Psychol and Psyc.* 2004;9(2):271-288.

23. Kuhlthau K, Luff D, Delahaye J, et al. Health-Related Quality of Life of Adolescent and Young Adult Survivors of Central Nervous System Tumors: Identifying Domains From a Survivor Perspective. *J of Ped Oncol Nurs.* 2015;32(6):385-393.

24. Kent EE, Parry C, Montoya MJ, et al. “You're Too Young For This”: Adolescent and Young Adults' Perspectives on Cancer Survivorship. *J of Psychosocial Oncol.* 2012;30(2):260-279.

25. Kumar AR, Schapira L. The Impact of Intrapersonal, Interpersonal and Community Factors on the Identity Formation of Young Adults with Cancer: A Qualitative Study. *Psycho–Oncology.* 2013;22(8):1753-1758.

26. Hammond V. My Lost Survivor. *Narr Inq in Bioethics.* 2014;4(1):E4-6.

27. Carlson-Green B. Brain Tumour Survivors Speak Out. *J of Ped Oncol Nurs.* 2009;26(5):266-279.

28. Crom DB. “I Think You Are Pretty; I Don't Know Why Everyone Can't See That”: Reflections From A Young Adult Brain Tumor Survivor Camp. *J of Clin Oncol*. 2009;27(19):3259-3261.

29. Boydell KM, Stasiulis E, Greenberg M, Greenberg C, Speigler B. I’ll Show Them: The Social Construction of (In)Competence in Survivors of Childhood Bain Tumours. *J of Ped Oncol Nurs.* 2008;25(3):164-174.

30. Cavers D, Hacking B, Erridge S, et al. Adjustment and support needs of glioma patients and their relatives: serial interviews. *Psycho-Oncology.* 2012;22(6):1299-305.

31. Chen C, Chen Y, Haase JE. Games of Lives in Surviving Childhood Brain Tumors. *Western Journal of Nurs Res.* 2008;30(4):435-457.

32. Gately L, McLachlan SA, Dowling A, Philip J. Surviving Glioblastoma, A Sense of Disconnection. *J of Clin Neurosci.* 2020;81:284-289.

33. Nicklin E, Pointon L, Glaser A, et al. Unmet support needs in teenage and young adult childhood brain tumour survivors and their caregivers: “it’s all the aftermath, and then you’re forgotten about”. *Support Care Cancer*. 2021;29:6315–6324.

34. Rabelais E, Jones NL, Ulrich CM, Deatrick JA. Meaning Making and Religious Engagement Among Survivors of Childhood Brain Tumours and their Caregivers. *Oncol Nurs Forum.* 2019;46(2):170-184

35. Simpson J, Heath J, Wall G. Living with a Pituitary Tumour: A Narrative Analysis. *Psychol and Health.* 2014;29(2):162-176.

36. Zwiers A, Campbell C, Evans M, Kirkwood K. Constructing the Meaning of Survivor With Former Pediatric Brain Tumour Patients. *J of Ped Oncol Nurs.* 2015;32(3):143-152.

37. Davies E, Hall S, Clarke C. Two Year Survival Ater Malignant Cerebral Glioma: Patient and Relative Reports of Handicap, Psychiatric Symptoms and Rehabilitation*. Disability & Rehabilitation.* 2003;25(6):259-266.

38. Gunn ME, Mort S, Arola M, et al. Quality of Life and Late-Effects Among Childhood Brain Tumour Survivors: A Mixed Method Analysis. *Psycho-Oncology.* 2016;*25:*677-683.

39. Liptak C, Brinkman T, Bronson A, et al. A social Programme for Adolescent and Young Adult Survivors of Pediatric Brain Tumours: The Power of a Shared Experience. *J of Psychosoc Oncol.* 2016;34(6):493-511.

40. Lucas MS, Barakat LP, Jones NL, Ulrich CM, Deatrick JA. Expectations For Function And Independence By Childhood Brain Tumours. *Narr Inq in Bioethics.* 2014;4(3):233-251.

41. Lucas MS, Barakat LP, Ulrich CM, Jones NL, Deatrick JA. Mother-Caregiver Expectations for Function Among Survivors of Childhood Brain Tumours. *Support Care Cancer.* 2016;24(5):2147-2154.

42. Hocking MC, Quast LF, Brodsky C, Deatrick JA. Caregiver perspectives on the social competence of pediatric brain tumour survivors. *Support Care Cancer.* 2017;25(12):3749-57.

43. Liaset IF, Kvam L. Experiences of Returning to Work After Brain Tumour Treatment. *Work.* 2018;60(4):603-612.

44. Deatrick JA, Barakat LP, Knafl GJ, et al. Patterns of Family Management for Adolescent and Young Adult Brain Tumor Survivors. *J of Family Psychology.* 2018;32(3):321- 332.

45. Palma E, Deatrick JA, Hobbie WL, et al. Maternal Caregiving Demands for Adolescents and Young Adult Survivors of Pediatric Brain Tumours. *Oncol Nurs Forum.* 2015;42(3):222-229.

46. Bonanno M, Bourque CJ, Aramideh J, et al. Articulating viewpoints to better define and respond to the needs of adolescents and young adult survivors of pediatric brain tumors. *J Psychosoc Oncol*. 2022;40(3):347-365.

47. Desjardins L, Young M, Hancock K, et al. Pediatric Brain Tumor Survivors' Understanding of Friendships: A Qualitative Analysis of ADOS-2 Interview Responses. *J Pediatr Psychol*. 2022;47(6):662-673.

48. Eiser C, Vance YH, Horne B, et al. The value of the PedsQLTM in Assessing Quality of Life in Survivors of Childhood Cancer. *Child Care Health and Development.* 2003;29(2):95-102.

49. Palmer S, Mitchell A, Thompson K, Sexton M. Unmet Needs Among Adolescent Cancer Patients: A pilot study. *Palliative and Supportive Care.* 2007;5(2):127-34.

50. Barakat LP, Li Y, Hobbie WL, et al. Health-Related Quality of Life of Adolescent and Young Adult Survivors of Childhood Brain Tumours. *Psycho-Oncology.* 2015;24:804-811.

51. Macartney G, Harrison MB, VanDenKerkhof E, Stacey D, McCarthy P. Quality of life and Symptoms in Pediatric Brain Tumour Survivors: A Systematic Review. *J of Ped Oncol Nursing.* 2014;31(2):65-77.

52. Hall AE, Boyes AW, Bowman J, et al. Young adult cancer survivors’ psychosocial well-being: a cross-sectional study assessing quality of life, unmet needs, and health behaviours. *Support Care Cancer* 2012;20(6):1333-41.

53. Dixon-Woods M, Sutton AJ, Shaw RL, et al. Appraising Qualitative Research for Inclusion in Systematic Reviews: A Quantitative and Qualtitative Comparison of Three Methods. *J of Health Sci.* 2007;12(1):42-47.
